# Transdermal water-in-oil nanocarriers of nitric oxide for triggering penile erection

**DOI:** 10.1038/s41598-018-25786-x

**Published:** 2018-05-09

**Authors:** Eunryel Nam, Saejong Yoo, Hwi-Yool Kim, Young-Rok Kim, Yun Jung Heo

**Affiliations:** 1 Department of Mechanical Engineering, College of Engineering, Kyung Hee University, Yongin, 17104 Republic of Korea; 2 Department of Veterinary Surgery, College of Veterinary Medicine, Konkuk University, Seoul, 05029 Republic of Korea; 3 Department of Food Science and Biotechnology, College of Life Sciences, Kyung Hee University, Yongin, 17104 Republic of Korea

## Abstract

Men’s sexual health can have significant effects on a man’s self-esteem, sexual relationship and male reproductive functions. Although commercially available drugs (e.g., VIAGRA and CIALIS) show effective treatment of erectile dysfunction (ED), patients with severe ED fail to respond to these medicines. Topical nitric-oxide (NO) delivery to penis can be a painless, alternative solution with severe ED because NO triggers erection and diffuses to the trabecular arteries and smooth muscles in the penis. We here develop water-in-oil (W/O) nanoemulsions (NEs) that contain NO and can directly spread on the penis. We optimize NE formation conditions including hydrophilic-lipophilic balance (HLB) and ratio of oil, water and surfactants. Then, by spreading NEs on penis skin of intact middle aged dogs, we verify medication effects and safety of the NEs *in vivo*. The water-in-oil NEs can be a promising non-invasive medication for ED patients with low response to a phosphodiesterase type 5 (PDE5) inhibitor, thus increasing quality of life in the aging society.

## Introduction

Sexual health is crucial to personal and social values due to the quality of life and reproductive function. Erectile dysfunction (ED) is defined as limited tumescent penis and inability to maintain stiffness of the penis for satisfactory sexual intercourse. ED has been gathering attention because ED affects more than 150 million men worldwide and is caused by various psychological and physiological factors^1^, especially smoking^2^, aging^3^, diabetes^4^ and cardiovascular diseases^5^. Most research efforts and remedies have focused on a phophodiesterase type 5 (PDE5) inhibitor (e.g., VIAGRA and CIALIS). PDE5 normally degrades 3′, 5′-cyclic guanosine monophosphate (cGMP) that is the second messenger molecule, which induces dilation of trabecular arteries and relaxation of corporeal smooth muscle cGMP. The PDE5 inhibitor obstructs the reduction of cGMP and raises the level of cGMP. These processes finally bring about blood-filling in the corpora cavernosa, and then erection occurs^6^. However, patients with severe ED caused by aging, deterioration of endothelium dysfunction and unrecognized hypogonadism fail to respond to a PDE5 inhibitor medicines^7^. Nevertheless, retained cGMP level can induce the erection by continuous supply of nitric oxide (NO); NO is a trigger of erection and diffuses to the trabecular arteries and smooth muscles in the penis. The increased level of nitric oxide stimulates the production and accumulation of cGMP by binding to soluble guanylate cyclase. Finally, NO leads the penile erection. Provided that NO is transdermally delivered to the erectile tissue through skin by non-invasive topical medication, the medication can be a painless, convenient ED treatment without serious side effects.

Nanoemulsion (NE) is widely used as an effective transdermal delivery system in pharmaceutics and cosmetic industry because it possesses a large surface area and high free energy in comparison to macro-scale emulsions^8^. Although the size of emulsions is usually larger than intercellular gap, nanoparticles with tens of nanometer diameter are known to enter dermis through various pathways such as hair follicles and glands^9^. Moreover, below 10 nm nanoparticles can easily penetrate skin and extend into the deeper skin layers than sub-micron particles whose size is from 100 nm to 1 μm^10^. Consequently, we formulated NE with size less than 10 nm to deliver NO through skin.

**Figure 1:**
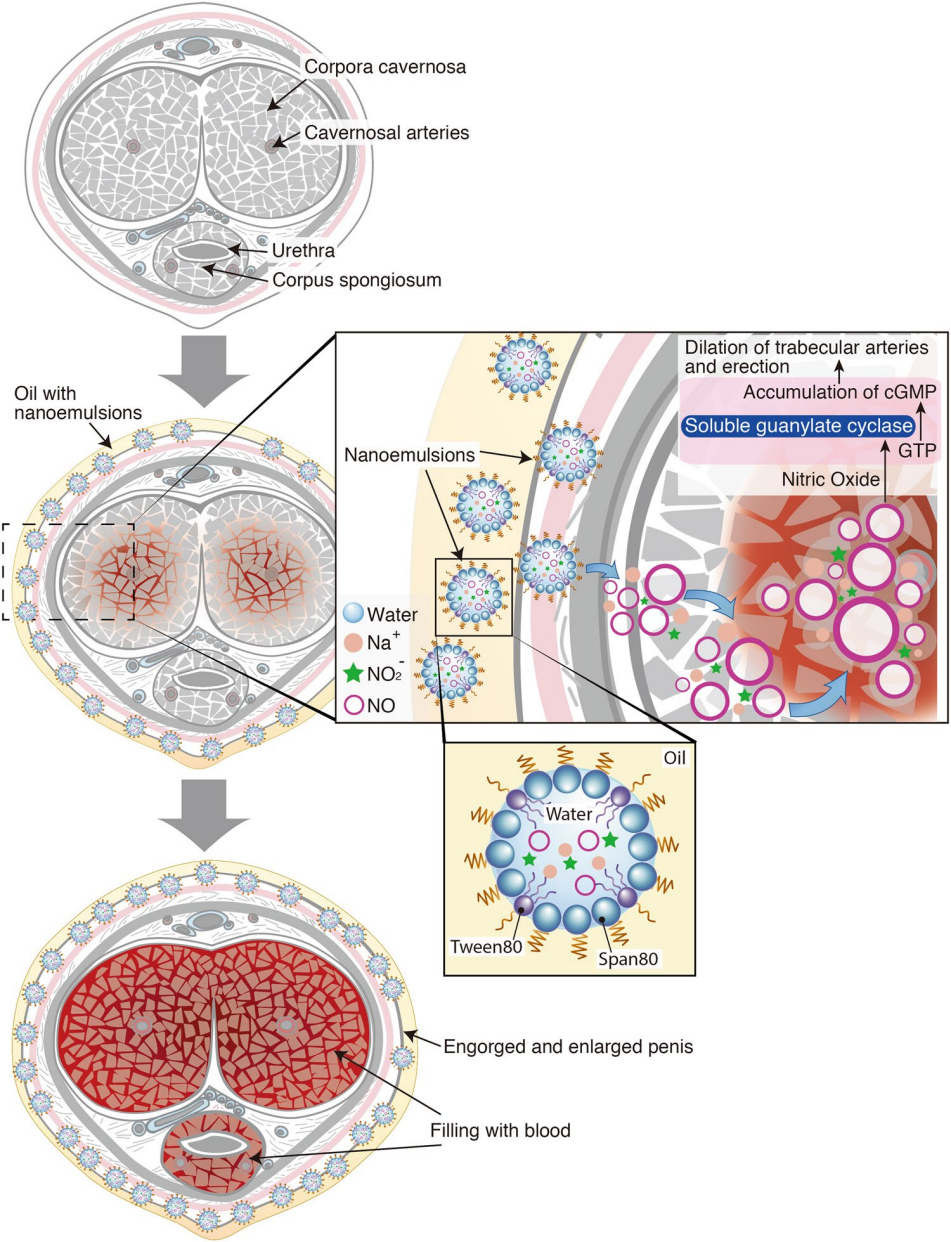
The NE with nitric oxide which is locally applied on the penis transports NO into the corpora cavernosa. The NO released from the NE binds soluble guanylate cylclase and increases the level of cGMP, leading to the dilation of trabecular arteries and relaxation of corporeal smooth muscle. The corpora cavernosa is then filled with blood and erection occurs.

Figure 1 shows mechanism of penile erection by means of the nitric oxide in the W/O NE. Upon application of the NE dispersed in an oil base onto the penis, NO_x_ is readily transported through skin to the fibrous tissue surrounding the corpora cavernosa. The NO works as a trigger of penile-erection accompanying engorgement and enlargement of penis due to repletion of blood in the corpora cavernosa.

We here develop water-in-oil (W/O) NE that contain NO and can directly apply on the penis. We optimized NE formation conditions including hydrophilic-lipophilic balance (HLB) and ratio of oil, water and surfactants. Then, by spreading the NEs on penis skin of intact middle-aged dogs, we verified medication effects and safety of the NEs *in vivo*.

## Results and Discussion

### Fabrication of nanoemulsions with nitric oxide

**Figure 2:**
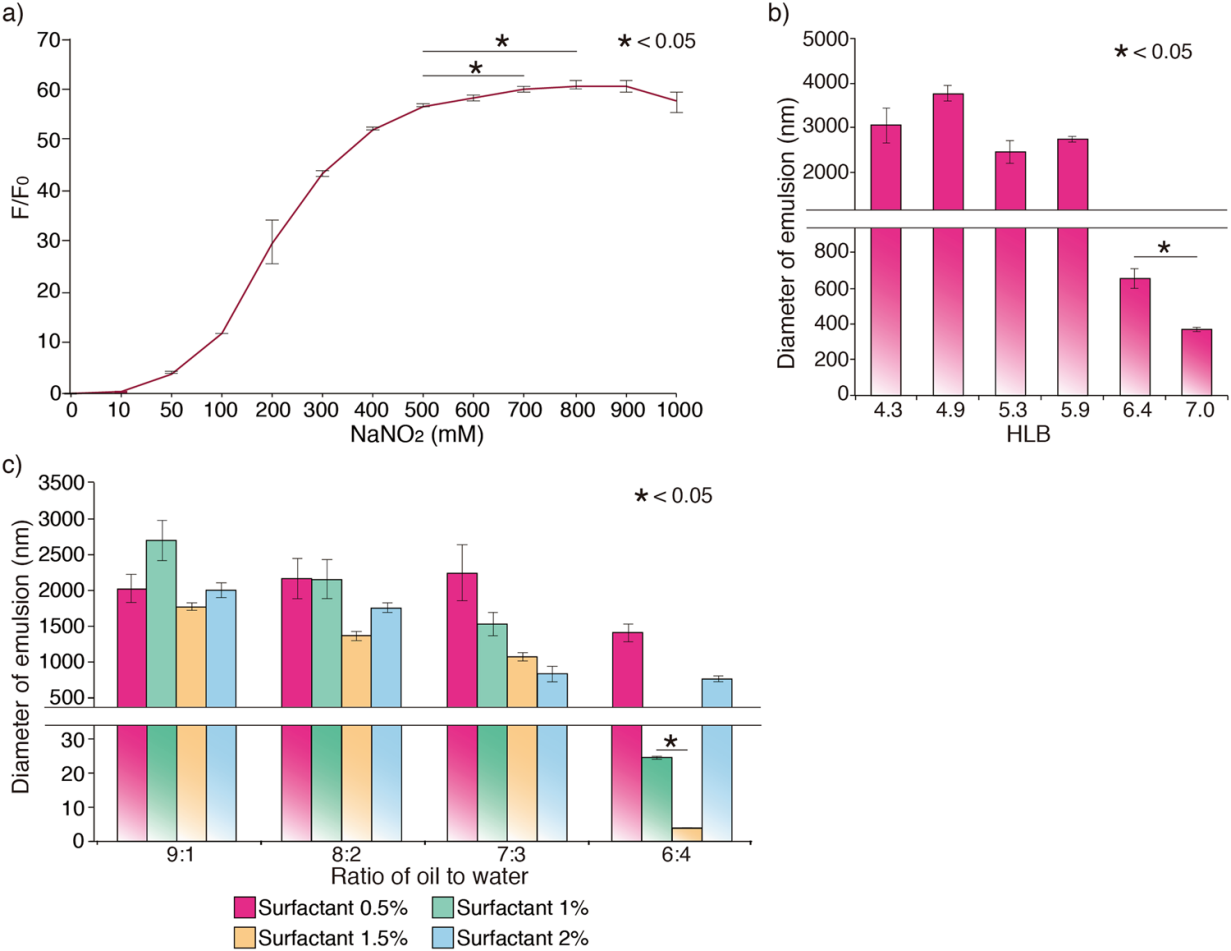
(**a**) Creation of NO in various concentration of NaNO_2_ solution. Amount of NO increased until 500 mM NaNO_2_ and reached a plateau at 800 mM (F_0_: Fluorescence intensity at 0 mM NaNO_2_ solution). (**b**) The size of emulsions prepared with varying HLB value. HLB value of 7 produced the smallest emulsions among the tested HLB values ranging from 4 to 7. (**c**) Diameter of emulsions at different mixture ratio of surfactant, oil and water. The NE was fabricated by mixtures of oil and water in the ratio 6: 4 with 1.5% (v/v) surfactant.

To determine the effective concentration of NaNO_2_ for W/O NE, we compared relative ratio of NO in various densities of NaNO_2_ solution using DAR-2 that is the marker of NO. Fluorescence intensity indicating the concentration of NO steeply increases at 100–300 mM and saturated at over 800 mM of NaNO_2_ (Fig. 2a). Thus, we used NaNO_2_ concentration of 800 mM for carrying maximum amount of NO.

We also found effective hydrophilic-lipophilic balance (HLB) of surfactant and the ratio of oil, water and surfactant; they are key factors for the preparation of emulsions in nano-size. HLB value is related with properties of NEs that could decide formulation of oil-in-water or water-in-oil NEs. In this classification, water-in-oil NEs are developed under 7 of HLB value. According to this system, we changed ratio of hydrophilic and lipophilic surfactants to make appropriate water-in-oil NEs and find the value that could formulate the smallest size of transdermal NEs^11^. Firstly, we investigated the size of emulsions with varying HLB values to determine an optimum HLB value. Because the NO in NaNO_2_ solution is transported as a W/O NE, we measured the size of emulsion produced with HLB ranging from 4 to 7, which is known to be appropriate for W/O NE. At HLB value below 6, the diameter of emulsions was shown to be above 2 μm. At HLB value of 7, the diameter decreased significantly comparing with that of the emulsions with less than HLB value of 6 (Fig. 2b). Since we aim to develop the percutaneously deliverable NEs that can penetrate skin and deliver NO inside the penis, we used HLB value of 7 to fabricate nanosize emulsions in this study. Secondly, to determine the ratio of oil, water, and surfactant, we mixed the three with various ratios. Since surfactant controls surface tension between water and oil, volume of surfactant greatly influence the size of NEs and stability^12^. Moreover, the ratio of water and oil is also closely related to formulate stable NEs^13^. To find proper the ratio of water and oil that surfactant can make the small size of NEs, the diameter of NEs were compared with each others that are formulated at the different ratios of water and oil and volume of surfactant. Mixtures of oil and water in the ratio of 6:4 showed the smallest emulsions for surfactant concentration of 0. 5–2% (v/v). We found that the surfactant concentration of 1.5% with the same ratio of oil and water (6:4) dramatically decreased the diameter of emulsion down to 5 nm (Fig. 2c and Table S1). This result indicates that concentration of surfactant is more important than the ratio of oil and water in fabrication of emulsions in nano-scale. Taken together, we developed NE of ~5 nm that can easily penetrate skin and extend into the deeper skin layers of penis.

**Figure 3:**
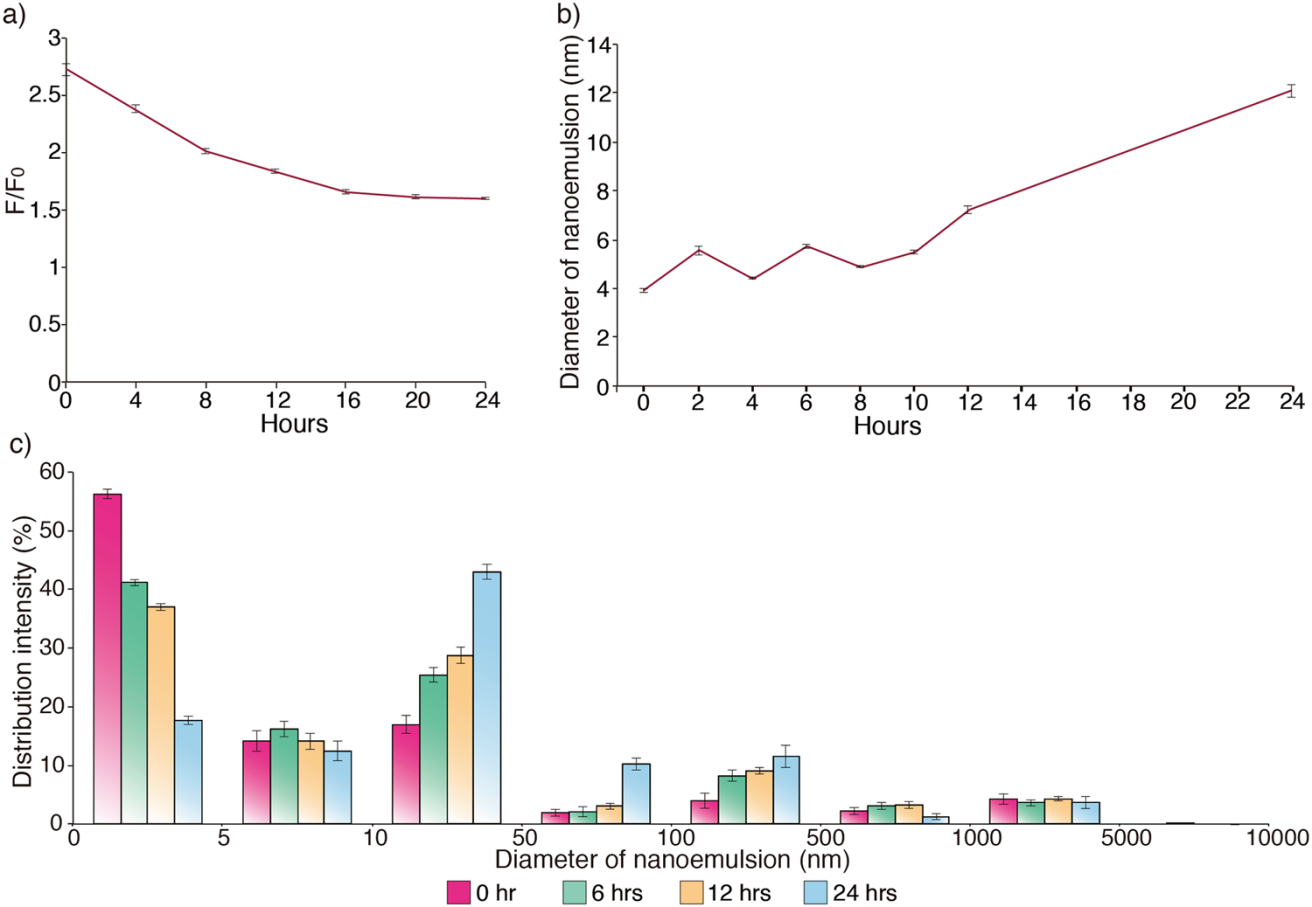
(**a**) Content of NO in the NE for 24 hours. The fluorescence intensity of NO slowly decreased over 24 h of storage. (F_0_: Fluorescence intensity of the NE without NO). (**b**) Changes in diameter of the NE for 24 hours. The diameter of NE started to increase from 10 h after fabrication, resulting in 3-fold increase after 24 h of storage. (**c**) Distribution intensity in the size of NE over the course of storage time.

To prove that the NEs can effectively carry NO, we evaluated the content of NO in NEs for 24 hours. Fluorescence intensity of the NEs with 800 mM NaNO_2_ was approximately 2.7 times higher than that of the NEs without NO right after emulsion formation, and slowly decreased to 1.5 in 16 hours (Fig. 3a), of which the intensity remained constant until 24 h, indicating that the NEs contain NO for at least 24 hours. To define stability of the NEs, the diameter of NE was measured for 24 hours. The NE diameters less than 6 nm was maintained for 10 hours from emulsion formation (Fig. 3b). However, the diameter of NE gradually increased with time and reached to ~12 nm that is three times bigger than the NE immediately after formation.

Although the size of NEs fluctuated for 10 hours, difference in size is less than 2 nm. After over 12 hours of formulation of NE, distribution intensity from 0 to 5 nm diameter of NEs decreased and the intensity from 10 to 500 was increased (Fig. 3c). Aggregation of NE, increases diameter of NEs, show unstability of NEs. Since size of nanoemulsions in this work showed an increasing tendency after 10 hours, we concluded that the NEs showed good stability for 10 hours based on size change in NEs for 24 hours. Further work on finding biocompatible additives can provide good stability for long term that is crucial for practical use.

### Animal experiments

#### Penile erection in dogs under anesthesia

We then experimentally proved that our NEs could be applied for erectile dysfunction treatment through animal tests. We here used two 7-year and two 8-year old intact male beagle dogs. We used dogs with 7–8 years old that are equivalent to middle aged human; approximately 40% of men at age of 40 s suffer with erectile dysfunction. We applied the oil with NE under anesthesia to minimize other factors such as over-excitement, blood flow by dog’s movement and rubbing, which could affect the erection activity of tested animal. We also took a blood samples from the corpus cavernosum under anesthesia. We exposed penis from prepuce to directly apply the NEs on penile mucous. The penile mucous in canine is equivalent to a foreskin of penis in human. However, penis is covered up with prepuce in dogs. The prepuce is covered with thick hair on several millimeters of thick skin in canine, whereas foreskin of human has no hair and thin skin with thickness of approximately 546 μm^14^. Furthermore, cavity exists between the prepuce and penis in canine. Since the prepuce hinder the direct apply of NEs, we exposed the penis from the prepuce.

We evaluated penile erection based on the diameter, redness, and blood NOx concentration in penis. The length, diameter, and rigidity of penis are regarded as typical erection indicators in human^15^. In this study, we only evaluated the diameter and redness of canine penis because of anatomical difference in penis between human and canine; the canine penis has a narrow bone that helps penis penetrate the vagina without erection. Since penis bone could cause misdiagnosis in stiffness, we excluded the rigidity of penis.

**Figure 4:**
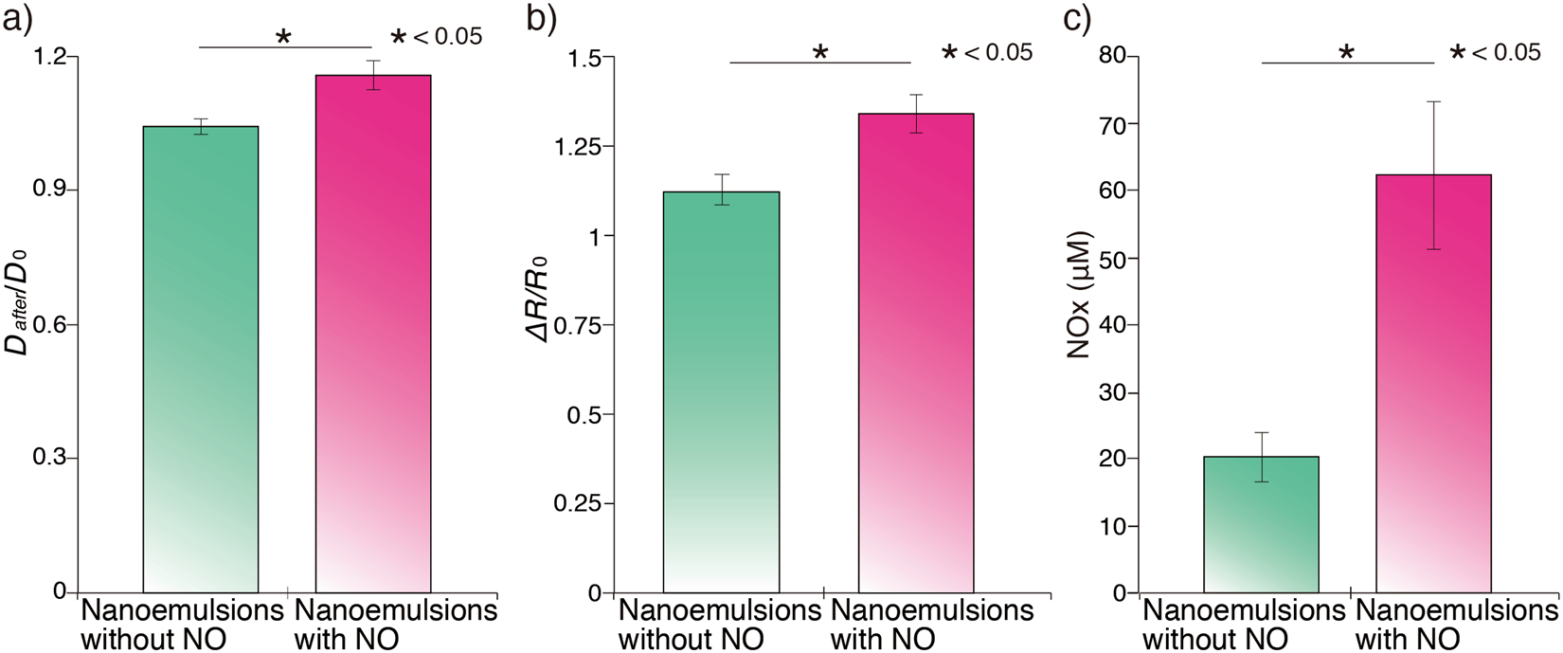
(**a**) Diameter of the penis after application of the NE with NO under anesthesia. The diameter of penis after application of the NE with NO increased significantly comparing with the control group treated with the oil without NO (D_0_: Diameter of penis without application of oil). (**b**) Redness value of the penis after application of the NE with NO under anesthesia. The redness value after application of the NE with NO was significantly higher than the control group (R_0_: Redness value of penis without application of oil). (**c**) Blood NOx concentration after application of the NE with NO. The level of blood NOx concentration increased 3-fold after the application of the NE with NO compared with control group.

To evaluate the enlargement of penis, we measured diameter of penis (Fig. 4a). After applying NE with NO on penis skin, diameter of penis increased 15% comparing with that before NE application. No such increase in diameter was observed in negative control groups (Fig. 4a). There is a study reporting that the penis diameter of average canine (mean age: 4.44 years old) increased 32.8% during erection^16^. As mentioned earlier approximately 40% of men at age of 40 s suffered with erectile dysfunction. Moreover, prevalence of the erectile dysfunction rises almost 10% as increase of age in middle age^17^. Aging also affects diameter of the penis and the corpus cavernosum in dogs^16^. Relatively low degree of enlargement in penis diameter in this study comparing with the previous report would be explained by the fact that we used dogs with 7–8 year-old that is equivalent to middle ages in human.

Then, we quantified redness of the penis to evaluate dilation of vessels on the mucous membrane. While the penis after applying NEs without NO showed slightly higher degree of redness than that of the penis exposed from the prepuce, the one treated with NEs containing NO showed dark red surface with distinguishable dilated-vessels, which are clear indicatives of erection (Fig. S1a). The redness of membrane increased approximately 35% and 12% after applying NE with NO and those without NO, respectively, in comparison to the penis exposed from the prepuce (Fig. 4b). Thus, direct application of NE to the mucous membrane on penis was effective for ED medication in dogs. We found that NE without NO can increase redness in the penis mainly due to irritation of mucous tissue. The penis exposed from the prepuce and directly contacted with air. This exposure could irritate the mucous membrane of the penis that is normally covered up with and protected by the prepuce.

Vasodilation and increase in diameter of penis were observed in dogs after applying NEs with NO. To verify that NO in NEs penetrated skin and caused erections in dogs, blood was collected from the penis after applying NEs with NO. Thus, we measured blood nitrite/nitrate oxide (NOx) level using the nitric oxide assay with the Griess reagents that allows only for measuring the concentration of NOx including NO_2_^−^ and NO_3_^−^. The level of NOx was quantified in penis blood from the corpus cavernosum after spreading the NE with NO on penis skin. On the other hand, blood from jugular vein was collected from the group treated with NEs without NO because we could not extract enough amount of blood for NOx assay from the penis without erection. The blood NOx level after application of the NE with NO was approximately 3 times higher than that of a control group treated with NE without NO (Fig. 4c). Although the site of blood gathering was different, shortage of blood in the penis after application of the NE without NO indicates that the erection did not occur in the absence of NO. The blood NOx concentration after applying NE without NO was similar to the average blood NOx level in canine^18^. From the results, NO in the NEs is successfully transported into the penis and caused erection.

Methemoglobinemia, high levels of methemoglobin, is commonly considered as a side effect of nitric oxide. The methemoglobin that is caused by elevated NOx concentration in blood hinders transport of oxygen by hemoglobin^19^. Above 1% methemoglobin level leads to cyanosis and dypsnea^20^. In infants having slightly higher blood nitrates concentration, blood nitrates level was 130 μM when below 1% of methemoglobin existed. The blood NOx concentration after applying NEs with NO was much less than the blood nitrates level that cause methemoglobinemia. The dogs after application of the NE with NO showed no clinical sign of methemoglobin including cyanosis in penis, dyspnea, and systemic side effects such as increase or decrease of heart rate. Consequently, the elevated NOx level after application of the NE with NO is safe and only affects penile erection. Further safety investigation (e.g., tissue sectioning and image analysis using alternative animal experiments) can strengthen our conclusion^21^.

#### Applied NE to dogs with consciousness

**Figure 5:**
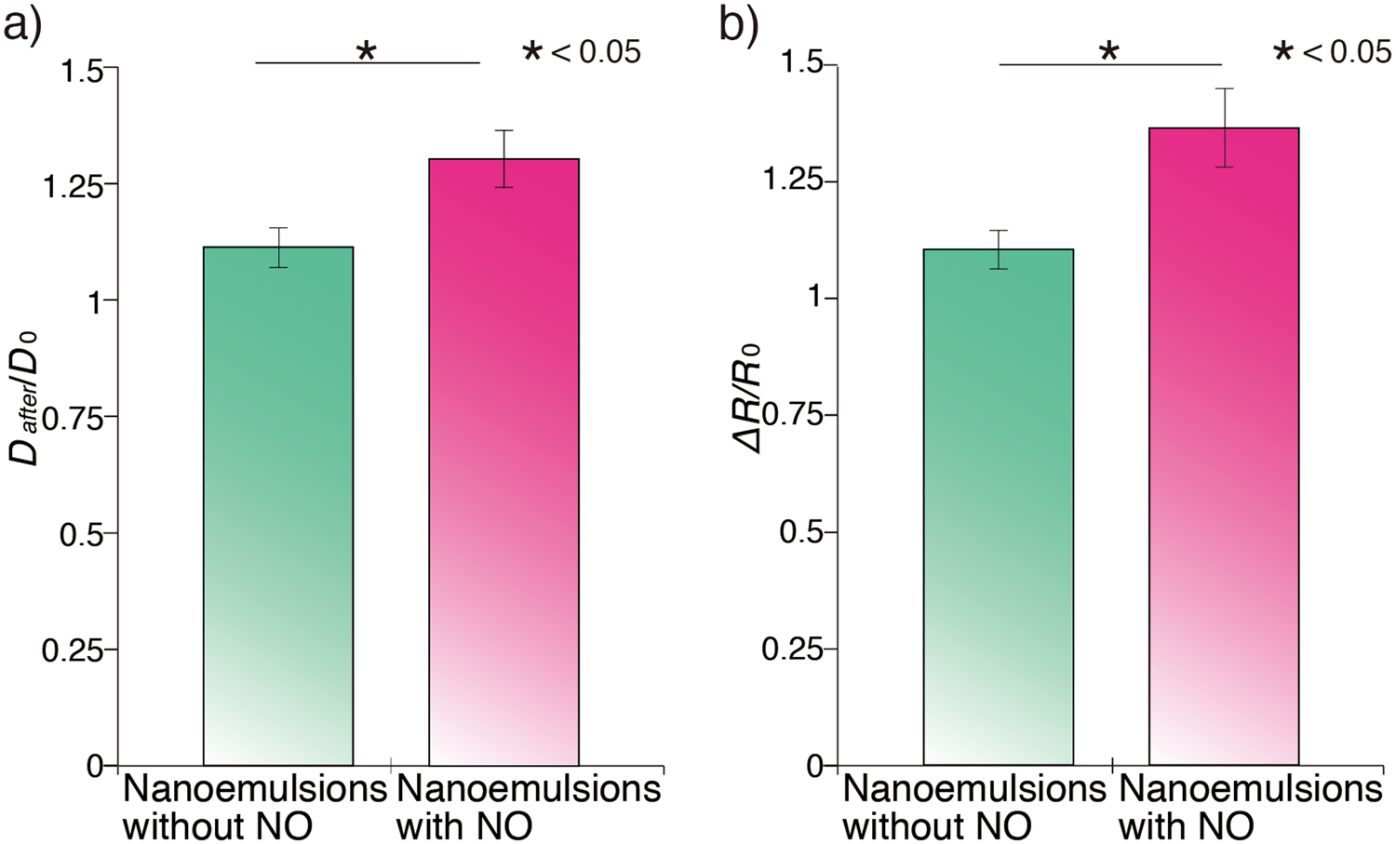
(**a**) Diameter change of the penis after application of the NE without and with NO. The increase of diameter after application of the NE with NO was significantly higher than the penis treated with the oil without NO (D_0_: Diameter of penis without application of oil). (**b**) Redness value of the penis after application of the NE without and with NO (R_0_: Redness value of penis without application of oil).

To prove efficacy and easy application of the NE with NO, we applied the NE with, and those without NO to dogs that were conscious, respectively. The penis after application of the NE with NO was enlarged and showed elevated level of redness compared with the penis exposed from the prepuce, and after application of the NE without NO, respectively (Fig. S1b). Then, we measured diameter of the penis and redness. Diameter of penis increased approximately 33%, and 14% after applying NE with NO, and without NO, respectively, compared with the diameter of penis when we pulled it out from prepuce (Fig. 5a).

Moreover, the redness of penis increased approximately 37%, and 10% after applying NE with NO, and those without NO, respectively, compared with that when we pulled it out from the prepuce (Fig. 5b). Excitement and movement of dogs during NE application might be responsible for the increase in diameter and redness of the penis compared with the group tested under anesthesia. We could not measure blood NOx concentration because the dogs expressed severe pain after injection of needle in penis without anesthesia.

## Conclusion

We developed NEs with NO that could induce penile erection by local and transdermal application on penis skin. We found that over 800 mM of aqueous NaNO_2_ solution could release effective amount of NO to trigger erection. We could form water-in-oil NEs with diameter of <5 nm by optimizing HLB and ratio of oil, water and surfactants. When we spread the obtained NEs with NO on penis skin of the middle aged dogs, blood NOx concentration in the penis increased, leading to penile erection without any notable topical and systemic side effects. Therefore, we believe that the water-in-oil NEs can be a promising non-invasive medication for ED patients with low response to PDE5 inhibitor, thus increasing quality of life in the aging society.

## Material and Methods

### Nitric oxide detection in NaNO_2_ solution

Following serial concentration of NaNO_2_ solutions in 0.1 M PBS were prepared: 0, 10, 50, 100, 200, 300, 400, 500, 600, 700, 800, 900 and 1000 mM. The nitric oxide marker DAR-2 (abcam, UK) was mixed with the NaNO_2_ solutions 1: 2. The fluorescence intensity (550 nm excitation, 585 nm emission) was measured by microplate (Infinite® 200 PRO, Tecan, Switzerland). Each fluorescence intensity (F) was divided by the intensity of 0 mM NaNO_2_ solution (F_0_).

### Preparation of the nanoemulsions with nitric oxide

Preparation of surfactant is in Table S1. 10% each surfactant in total volume was mixed with vitamin E antioxidant body oil (Product No. 04800, Cococare, Dover, NJ) and NaNO_2_ solutions 8: 2. Firstly, NaNO_2_ solution was added dropwise to the surfactant during stirring at 1500 rpm on a hot-plate stirrer. Then, the oil was also provided dropwise while stirring at 1500 rpm after 10 minutes later adding NaNO_2_ solution. After 10 minutes of stirring, the sonication was applied to the formation at 25% amplitudes for 2 minutes by a probe sonicator (Vibra-Cell, 20 kHz, Sonics, Newtown, CT). The size of emulsions was measured using a dynamic light scattering (Zetasizer NaNO S, Malvern, UK). The water-in-oil emulsions were prepared as previously describe. The ratio of surfactant, oil, and NaNO_2_ solutions were in Table S2. After the formation of emulsions, the dynamic light scattering (Zetasizer NaNO S) was used to measure the size of emulsions. Detection nitric oxide in the oil with NEs, and stability test of the NEs with nitric oxide are described in SI Materials and Methods.

### Animal experiments

The animal experiments in this study were ethically approved by the institutional animal care and use committee of Konkuk university (Approval ID number: KU17113). All animal experiments were performed in accordance with “Guide for the Care and Use of Laboratory Animals” of Institute of Laboratory Animal Resources Commission on Life Sciences National Research Council, USA, and under the supervision of veterinarians. Two 7 years old and tow 8 years old intact male dogs and weighing about 10 kg, were used in this study.

### Inducement of penile erection in dogs under anesthesia

All of the dogs were anesthetized with propofol and isoflurane in oxygen after intubation. Under general anesthesia, the penis of dogs was pulled out from prepuce. Then, the diameter of the penis body which is the site of one-third from the apex of the penis was measured. The NEs containing with and without nitric oxide were applied on the penis for 5 minutes. After remove the oil on the penis, we measured the girth of penis body. Percentage of increase in the diameter of the penis was calculated that change of the diameter between before and after application of the oil (*D*_*after*_) was divided by the diameter of penis before application of the oil (*D*_0_). The blood was gathered from corpus cavernosum and jugular vein after application of the NEs with nitric oxide and without nitric oxide by 29 gauge needles, respectively.

### Inducement of penile erection in dogs with consciousness

The application of the oil with and without nitric oxide was performed as previously described in the dogs with consciousness. The blood was not collected form the dogs with consciousness.

### Evaluation of redness of the penis

The pictures of penis before and after application of the NEs were analyzed by ImageJ (NIH). After split the images into red-green-blue channels, the mean gray value in the penis was measured. Percentage of the redness of the penis was calculated that change of the gray value between before and after application of the oil (*ΔR*) was divided by the gray value of penis before application of the oil (*R*_0_).

### Nitric oxide assay

Nitric oxide assay was carried out using the blood from the penis and jugular vein and a colorimetric kit (ab65328, abcam). 0.1% sodium citrate that is anticoagulation was added to the blood sample. The serum collected from the blood samples was transported to 10 kD Spin column (ab93349, abcam) and centrifuged at 15,000 g for 10 minutes at 4 °C. The procedures of assay were performed following a manufacturer’s instructions. Briefly, deproteinized blood samples were reacted with nitrate reductase and and enzyme cofactor for 1 hour at room temperature. After addition of Griess reagents, the absorbance at 540 nm by the microplate reader (Infinite® 200 PRO) was measured to determine the amount of nitrate/nitrite.

### Statistical analysis

The mean and standard deviation values of all results in this work were calculated and analyzed by using Student’s *t*-test at a significance level of *P* < 0.05.

## Electronic supplementary material

Supplementary Information
