# Supplementary material for: Transdermal water-in-oil nanocarriers of nitric oxide for triggering penile erection
Source: Sci Rep. 2018 May 9;8:7312. doi: 10.1038/s41598-018-25786-x (PMC5943240; doi:10.1038/s41598-018-25786-x)
Supplement: Supplementary file 1 — Supplementary Information [file 41598_2018_25786_MOESM1_ESM.doc]

**Supplementary Information**

**Transdermal water-in-oil nanocarriers of nitric oxide for triggering penile erection**

Eunryel Nam, Saejong Yoo, Hwi-Yool Kim, Young-Rok Kim, and Yun Jung Heo*

**1. SI materials and methods**

**1.1. Preparation of surfactant.** Surfactant was prepared as following Table S1(1).

**Table S1.** HLB values of blended Span 80 and Tween 80.

| HLB | Ratio of Span 80 to Tween 80 |
| --- | --- |
| 4.3 | 100: 0 |
| 4.9 | 95: 5 |
| 5.3 | 90: 10 |
| 5.9 | 85: 15 |
| 6.4 | 80: 20 |
| 7.0 | 75: 25 |

**1.2. Determination of ratio of the surfactant, oil, and NaNO_2_ solutions.**

The surfactant, oil, and NaNO_2_ solutions were mixed by following Table S2 (1).

**Table S2.** The ratio surfactant, oil, and NaNO_2_ solutions for fabrication of emulsions.

| Surfactant | Ratio of oil to NaNO_2_ solutions | Ratio of surfactant, oil, and NaNO_2_ solutions |
| --- | --- | --- |
| 0.5% | 9: 1 | 0.05: 8.955: 0.995 |
|  | 8: 2 | 0.05: 7.96: 1.99 |
|  | 7: 3 | 0.05: 6.965: 2.985 |
|  | 6: 4 | 0.05: 5.97: 3.98 |
| 1% | 9: 1 | 0.1: 8.91: 0.99 |
|  | 8: 2 | 0.1: 7.92: 1.98 |
|  | 7: 3 | 0.1: 6.93: 2.97 |
|  | 6: 4 | 0.1: 5.94: 3.96 |
| 1.5% | 9: 1 | 0.15: 8.865: 0.985 |
|  | 8: 2 | 0.15: 7.88: 1.97 |
|  | 7: 3 | 0.15: 6.895: 2.955 |
|  | 6: 4 | 0.15: 5.91: 3.94 |
| 2% | 9: 1 | 0.2: 8.82: 0.98 |
|  | 8: 2 | 0.2: 7.84: 1.97 |
|  | 7: 3 | 0.2: 6.86: 2.94 |
|  | 6: 4 | 0.2: 5.88: 3.92 |

**1.3. Detection nitric oxide in the oil with nanoemulsions.**

The nanoemulsions were prepared as previously describe. 2.4M NaNO_2_ solutions was mixed with DAR-2 (abcam, UK) solution 1: 2. We took pictures the oil with nanoemulsions for 24 hour by a microscopy (Leica, Germany). The mean of fluorescence density was quantified using ImageJ (NIH, Bethesda, ML). Each fluorescence density (F) was divided by the density of nanoemulsions without nitric oxide (F_0_).

**1.4. Stability test of the nanoemulsions with nitric oxide.** The nanoemulsions were prepared as previously describe. The dynamic light scattering (Zetasizer NaNO S) was used to measure the size and the distribution of sizes of emulsions for 24 hours.

**2. Results**

**2.1. Penile erection in dogs.**

**
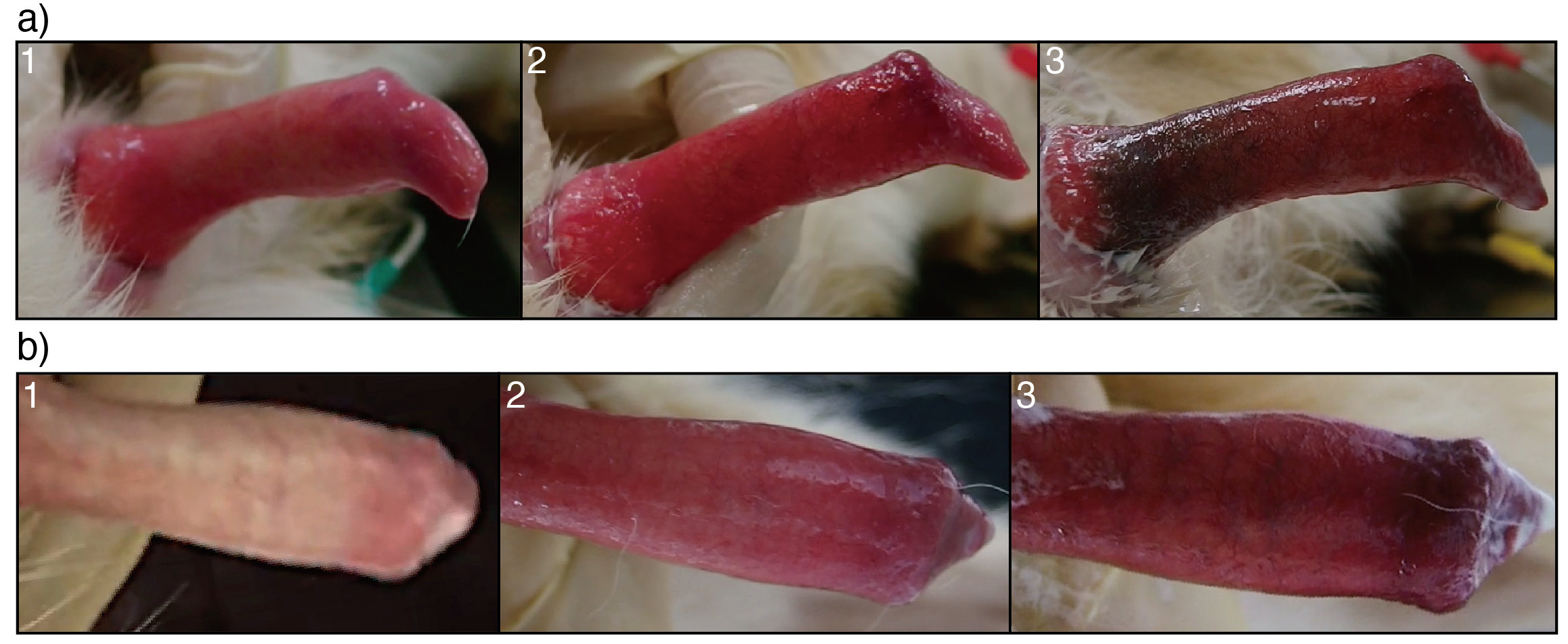
**

Fig. SI 1. (a) the canine penis without application of the oil (1), after application of the oil without NO (2), and application of the oil with NO (3) under anesthesia. The penis treated with NE with NO showed dark red surface and clearly distinguished dilated vessels (3). (b) the penis without application of the oil (1), after application of the oil without NO (2), and application of the oil with NO (3) in canine under consciousness.

1. H. Kunieda, and K. Shinoda, Evaluation of the hydrophile-lipophile balance (HLB) of nonionic surfactants. I. Multisurfactant systems*, J. Colloid. Interface. Sci***. 1**07, 107-121 (1985).
